# Supplementary material for: Impaired Development of Collagen Antibody-Induced Arthritis in Rab44-Deficient Mice
Source: Biomedicines. 2024 Nov 1;12(11):2504. doi: 10.3390/biomedicines12112504 (PMC11591669; doi:10.3390/biomedicines12112504)
Supplement: Supplementary file 1 [file biomedicines-12-02504-s001.zip › biomedicines-3239362-supplementary.pdf]

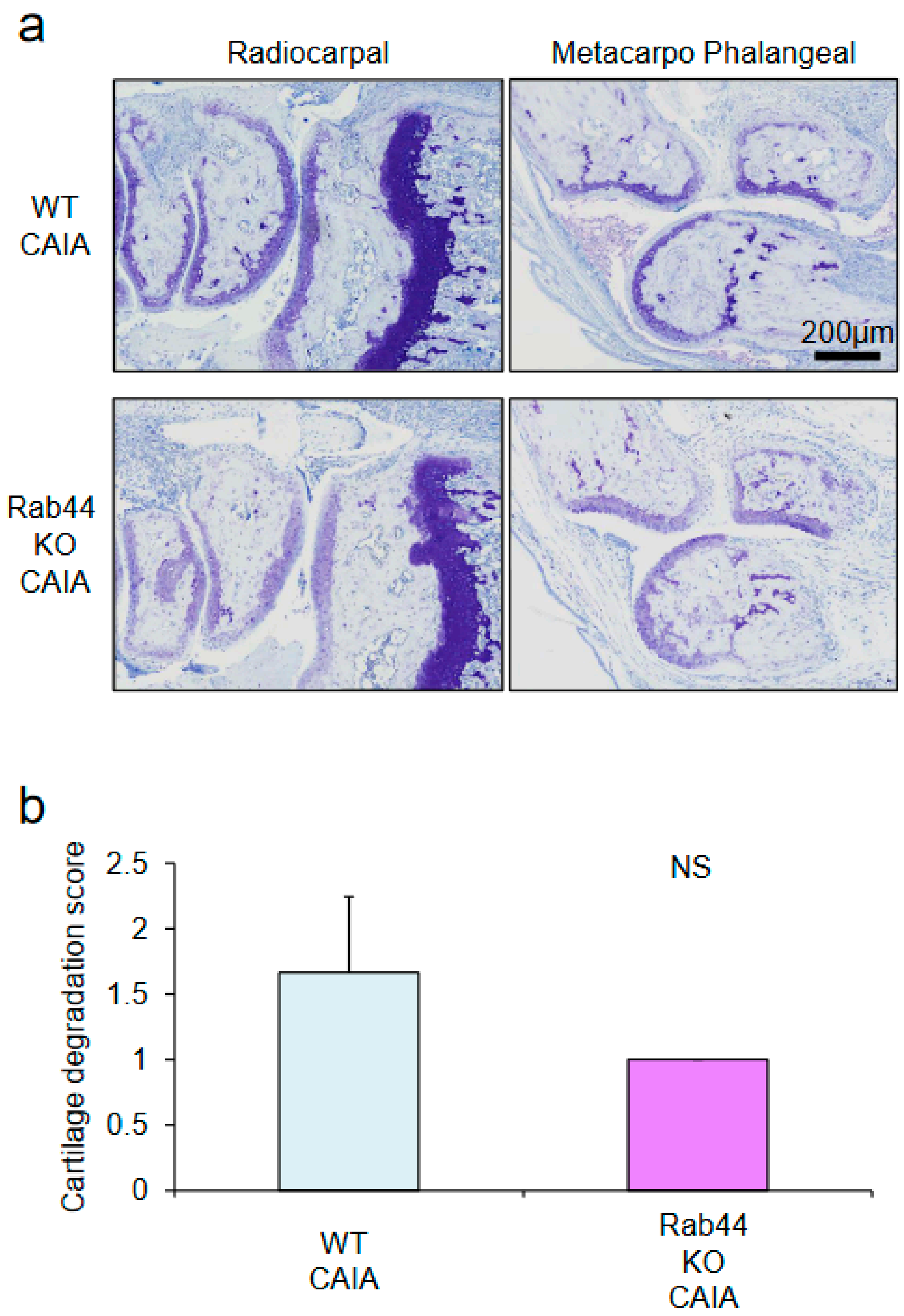

Figure S1. Histological analysis of the cartilage of radiocarpal joints of mice. Mitigation of histological cartilage degradation in CAIA mice in Rab44 knockout (KO) mice. (a) Toluidine blue (TB) staining of the radiocarpal and metacarpophalangeal joints in WT collagen antibody-induced arthritis (CAIA), and Rab44-KO CAIA mice, respectively. (b) Cartilage degradation scores were calculated from the average score of TB staining (*n* = 3); normal, score 0; mild loss of TB staining, score1; moderate loss of TB staining and cartilage loss, score 2; marked loss of TB staining with marked multifocal cartilage loss, score 3.

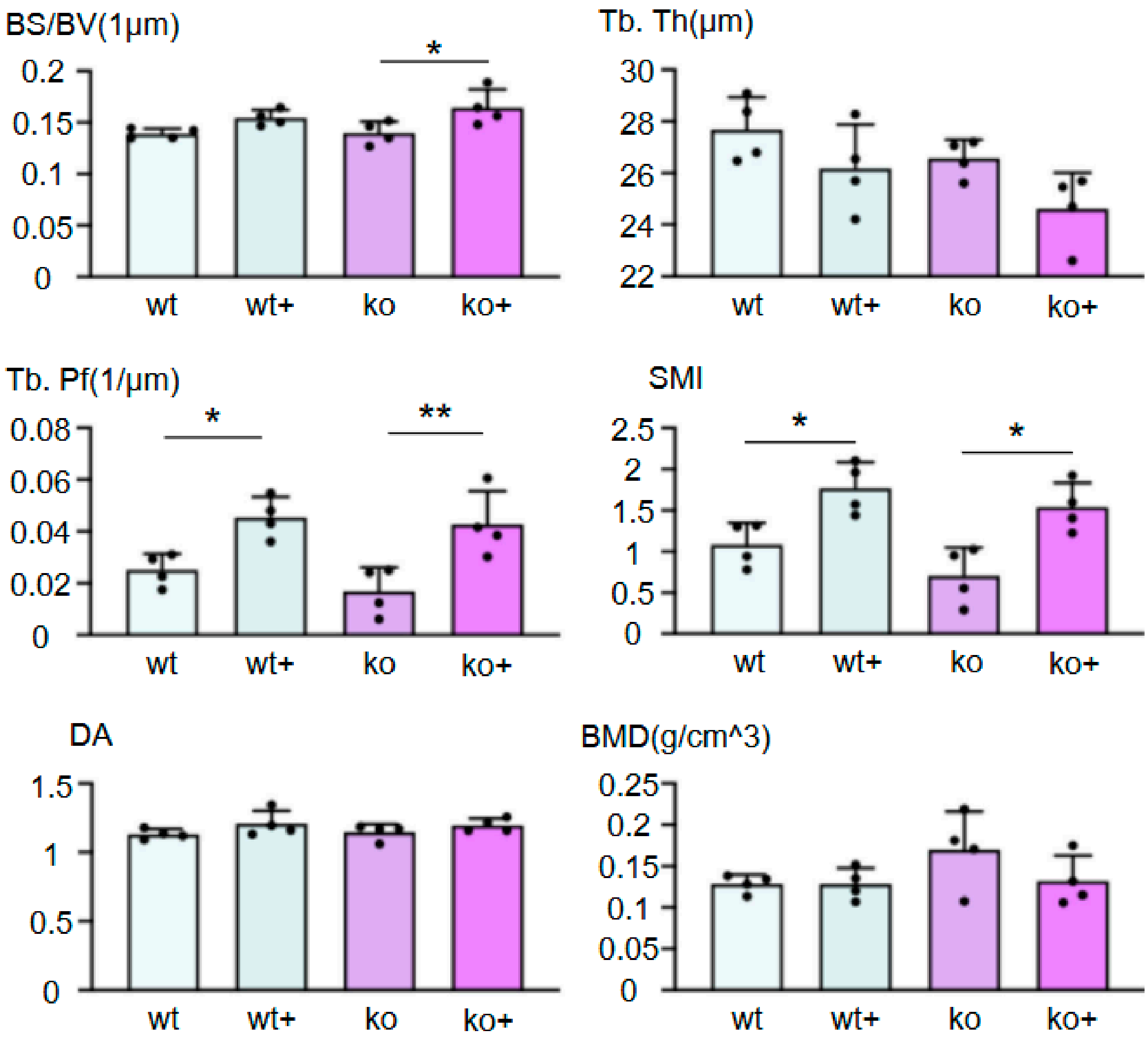

Figure S2. Micro-computed tomography (μ-CT) analysis of the trabecular bones of radiocarpal joints of mice. Remaining results from μ-CT analysis not shown in Fig. 5c are presented here. The evaluated bone parameters include the bone surface-to-volume ratio (BS/BV), trabecular thickness (Tb. Th), trabecular bone pattern (Tb. Pf), structure model index (SMI), degree of anisotropy (DA), and bone mineral density (BMD) in wild-type (WT) control (w-), WT collagen antibody-induced arthritis (CAIA) (w+), Rab44- KO control (k-), and Rab44- KO CAIA mice (k+) (n =4, *p < 0.05, **p < 0.01).
